# Cost-effectiveness of intensive inpatient treatments for severely obese children and adolescents in the Netherlands; a randomized controlled trial (HELIOS)

**DOI:** 10.1186/1471-2458-11-518

**Published:** 2011-06-30

**Authors:** Sabine Makkes, Jutka Halberstadt, Carry M Renders, Judith E Bosmans, Olga H van der Baan-Slootweg, Jacob C Seidell

**Affiliations:** 1 Department of Health Sciences, Faculty of Earth and Life Sciences, VU University Amsterdam, De Boelelaan 1085, Amsterdam, 1081 HV, the Netherlands; 2 Merem Treatment Centers, Heideheuvel, Soestdijkerstraatweg 129, Hilversum, 1213 VX, the Netherlands

## Abstract

**Background:**

Intensive combined lifestyle interventions are the recommended treatment for severely obese children and adolescents, but there is a lack of studies and their cost-effectiveness. The objective of this study is to compare the cost-effectiveness of two intensive one-year inpatient treatments and usual care for severely obese children and adolescents.

**Methods/Design:**

Participants are 40 children aged 8-13 and 40 adolescents aged 13-18 with severe obesity (SDS-BMI ≥ 3.0 or SDS-BMI ≥ 2.3 with obesity related co-morbidity). They will be randomized into two groups that will receive a comprehensive treatment program of 12 months that focuses on nutrition, physical activity and behavior change of the participant and their parents. The two programs are the same in total duration (12 months), but differ in inpatient treatment duration. Group A will participate in a 6 month intensive inpatient treatment program during weekdays, followed by six monthly return visits of 2 days. Group B will participate in a 2 month intensive inpatient treatment program during weekdays, followed by biweekly return visits of 2 days during the next four months, followed by six monthly return visits of 2 days. Several different health care professionals are involved, such as pediatricians, dieticians, psychologists, social workers, nurses and physiotherapists. Results will also be compared to a control group that receives usual care. The primary outcome is SDS-BMI. Secondary outcomes include quality of life using the EQ-5D and cardiovascular risk factors. Data will be collected at baseline and after 6, 12 and 24 months. An economic evaluation will be conducted alongside this study. Healthcare consumption will be based on actual resource use, using prospective data collection during 2 years through cost diaries. Quality Adjusted Life Years (QALYs) will be calculated using the EQ-5D.

**Discussion:**

This study will provide useful information on the effectiveness and cost-effectiveness of inpatient treatment in severely obese children and adolescents. Valuable information on long term effects, after 2 years, is also included.

**Trial registration:**

Netherlands Trial Register (NTR): NTR1678

## Background

In the Netherlands the prevalence of obesity in children and adolescents has increased during the last decades [1-3]. Generally, when an increase in prevalence is observed, there is a skewed distribution to the right instead of a normal distribution, which means that a disproportional large increase in the prevalence of severe obesity is observed [4]. The prevalence of obesity in children aged 4-15 years has increased from 0.2 to 2.6% in boys and from 0.5 to 3.3% in girls in the period 1980-2003 [3]. These trends are worrisome because obesity in children is associated with an increased risk of several chronic diseases such as diabetes type 2 and cardiovascular diseases and musculoskeletal, respiratory and psychosocial problems [5,6]. Obesity also tracks from childhood into adulthood and is predictive for significant health consequences in later life independent of adult BMI [7-9]. Besides having an adverse impact on healthy life years and quality of life, childhood obesity also has a substantial impact on health care utilization and results in a heavy financial burden for society [10,11]. Although many programs for the treatment of obesity exist, results have not been very promising, especially regarding their long term effectiveness. Nevertheless, a recent systematic review of inpatient programs for children showed greater reductions in the percentage overweight participants at post treatment and at follow-up compared with results from a recent meta-analysis of out-patient treatments [12]. Studies evaluating the effectiveness of treatment programs (with or without an inpatient period) aimed at children with severe obesity are relatively rare [12,13]. However, it has been suggested that ambulatory programs for severely obese children and adolescents are insufficiently effective and that there is a need for experienced, specialized pediatric obesity centers for intensive treatment by professionals with expertise in pediatric and adolescent medicine [14,15]. Spear et al. describe the need for comprehensive treatments that should be provided by multidisciplinary obesity care teams, including for example social workers, psychologists, dieticians, and exercise specialists [16]. The ideal approach in such comprehensive treatments would include dietary modification, an increase in physical activity, a reduction in sedentary activity and behavior modification [17,18]. A promising alternative for ambulatory care is inpatient treatment in specialized centers as mentioned above [12]. Heideheuvel (part of Merem Treatment Centers) is the only specialized clinic in the Netherlands offering an intensive combined lifestyle inpatient intervention, focusing on nutrition, physical activity and behavior change of the participants and their parents. This lifestyle intervention has a duration of 12 months, with a 6 month inpatient period and is designed for severely obese children and adolescents between 8 and 18 years. However such a lengthy inpatient treatment program involves high costs and a considerable burden for both the child and the family. Therefore, a new intensive combined lifestyle intervention also with a duration of 12 months, but with a shorter inpatient period (2 months) was developed at Heideheuvel with the aim of being equally effective but less costly and disturbing for family life.

The objective of this study is to compare the cost-effectiveness of these two intensive one-year inpatient treatments to each other and to usual care for severely obese children and adolescents.

## Methods/Design

### Design

**Figure 1:**
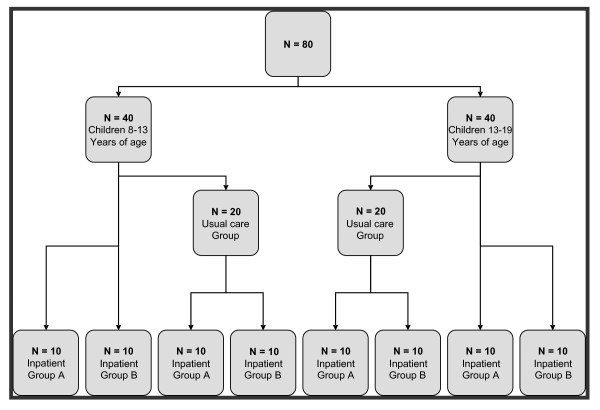
**Flow chart of participants**.

This study is designed as a randomized controlled clinical trial with three study arms. The cost-effectiveness of two intensive one-year treatments that vary in inpatient period length and usual care for severely obese children and adolescents will be evaluated. There is a one year follow-up after treatment. Participants who meet the inclusion criteria and consent to participate in the study will be randomized to one of the three study arms (described under 'interventions'). Because of the nature of the treatments evaluated in this study, parents and participants as well as professionals at Heideheuvel and in the usual care condition cannot be blinded to the type of intervention. A table of random numbers is used to randomize participants. At the beginning of the first year 40 (13-18 years) participants are randomized into: group A (10), group B (10) or group C (20). The participants in group C will be randomized into group A (10) or B (10) after one year of receiving usual care. This process is repeated for another 40 participants (8-13 years) after 6 months. At the end of the fourth year all 80 participants (40 in each inpatient treatment group) will have completed the program and 1 year of follow up (Figure 1). Data collection started in August 2009 and will continue until July 2013. The Medical Ethics committee (METc) of VU University Medical Centre approved the study design, protocol and informed consent procedures.

### Participants

The study population consists of children and adolescents aged 8-18 years referred to Heideheuvel by their own pediatrician. Pediatricians working at Heideheuvel screen the referred participants for eligibility to be included in the treatment. They must have a SDS-BMI ≥ 2.3 according to the growth curves based on the fourth Dutch National Growth Study of 1997 (this corresponds to the 99th percentile) and co-morbidity related to obesity (e.g. obstructive sleep apnea syndrome, raised insulin, diabetes type II, liver function disorders, dyslipidemia, worn out joints) or a SDS-BMI ≥ 3.0 (this corresponds to the 99.9th percentile). Participants will be excluded if they have syndromal or chromosomal determined obesity, obesity caused by endocrine disorders (hypothyroidism, Cushing syndrome, primary hyperinsulinemia, pseudohypoparathyroidism, acquired (structural) hypothalamic damage) or medicine use (e.g. oral steroids, antiepileptic drugs, antidepressants), severe psychiatric problems, an IQ below 75 or similar school level or if their parents are not willing to participate in the treatment. Written informed consents are obtained from both the participants and their parents.

### Interventions

The intensive combined lifestyle interventions focus on nutrition, physical activity and behavior change of the participants and their parents. Many disciplines are involved, such as pediatricians, dieticians, psychologists, social workers, nurses, physiotherapists, general exercise therapists and exercise therapists Cesar (a specific type of exercise therapy in The Netherlands that is mainly focused on posture, balance and coordination). Additional individual meetings with a psychologist, dietician or social worker are offered if participants, parents or professionals indicate that this is needed. During the inpatient period, children and adolescents will participate in an exercise program four times a week and nutrition education/behavior modification sessions once per week. Behavior modification topics include self-regulation, self-awareness, goal setting, stimulus control, coping skills training, cognitive behavior strategies and contingency management. Behavior modification is achieved through the '5 steps of problem solving'-plan. The first step is to define the problem and learn to describe it. The second step is to search for possible solutions for the defined problem. Step 3 is to make an inventory of the possible consequences for each possible solution described in the second step. The fourth step is to choose the best solution from the possible solutions. The last step then is to implement this solution and to evaluate this afterwards. If the problem is not solved, one can go back to the first step. The nutritional education component of the program will use an approach not primarily aimed at caloric restriction but rather at structured eating and healthier choices, focusing on improving the quality of the dietary intake, and on trying to establish a flexible control of eating behavior. During the weekends at home in the inpatient period the participants and their parents are required to accomplish exercises regarding nutrition and physical activity. During the period when the children are home again the learned behavior is practiced at all times.

Group A will participate in a 6 months intensive inpatient treatment program during weekdays, followed by six monthly return visits of 2 days. Group B will participate in a 2 month intensive inpatient treatment program during weekdays, followed by biweekly return visits of 2 days during the next four months, then followed by six monthly return visits of 2 days. During the inpatient treatment phase and the 2-day return visits all children and adolescents stay overnight at Heideheuvel. Parents of participants in groups A and B will attend 1 session every week during the inpatient period and after that 1 session per return visit of their child. Group C will receive usual care during one year, after which the participants will be randomly allocated to the groups A and B. This implies that during this year they remain under the care of their pediatrician and other health care professionals that might be involved in their treatment like the general practitioner, dietician, physiotherapist and psychologist.

### Detailed descriptions of treatments A and B

Treatment A already existed before the start of the study. Treatment B is based on treatment A, but includes a shorter inpatient period in the first half year to reduce the burden for parents and children. This expands the opportunity for implementation of the learned behavior at home. Treatment B also focuses more on involvement of the parents from the beginning.

The focus of both treatments is on knowledge and skills and implementation. In both treatments the same topics are covered during the educational sessions, but the form in which the education is given is slightly different (guidance by a psychologist, dietician or exercise therapist in treatment A and by group coaches in treatment B).

Both treatment A and B are divided into 4 phases: 1. assessment/knowledge acquisition phase (week 1-6/1-8), 2. Knowledge acquisition and skills building phase (week 7-18/9-18), 3. Implementation phase (week 19-26) and 4. Maintenance phase (27-52).

#### Phase 1 (weeks 1-6 for treatment A, weeks 1-8 for treatment B)

During the first phase extensive physical and psychosocial examinations are carried out. The children are medically examined and they have several individual appointments with among others the dietician and psychologist, both with and without their parents, to assess what the problem areas are for each child. During this period the children receive schooling at a study centre at Heideheuvel, except for the primary school children. They go to primary schools in the neighborhood where the teachers can monitor dietary habits of the children. A very important secondary goal in this phase is also the group formation.

#### Phase 2 (weeks 7-18 for treatment A, weeks 9-18 for treatment B)

In the second phase, there are weekly (group A) or biweekly (group B after the inpatient period) educational group sessions for the children with a psychologist covering different topics, such as dealing with emotions, self confidence and self image. During this period, the children of treatment A go to a school in the vicinity of Heideheuvel. Children of treatment B continue to receive schooling at a study centre at Heideheuvel during the inpatient period and the biweekly return visits, when they are home they go back to the school in their own neighborhood.

#### Phase 3 (weeks 19-26 for treatment A as well as for treatment B)

In the third phase, the knowledge acquired in the previous phases is put into practice. In weeks 19 and 20 the children of group A have the last sessions of the weekly education program supervised by a psychologist and group coach. The children of group B will have their last session in the last biweekly return visit. From week 20 on in group A, one weekly visit to a movement therapist can be exchanged for a visit to a sports centre in the vicinity of the clinic, if the children like to perform sports not possible at the clinic. The children of group B continue to do exercise during their biweekly return visit. During phase 3 the children in treatment A go to a school in the vicinity of Heideheuvel Children of treatment B continue to receive schooling at a study centre at Heideheuvel when they are there for their biweekly return visit, when they are home they go back to the school in their own neighborhood. Around week 22 the children in treatment A return to their families once for a maximum of 1 week. This is to practice the learned skills at home during a normal week and see which problems are encountered in their home environment. After this family leave the encountered problems are discussed and if necessary plans are adjusted before the children are going back home again.

#### Phase 4 (weeks 27-52 for treatment A as well as for treatment B)

In the fourth phase, the maintenance phase, the children live at home again and go to their own school. During this period, which is aimed at preserving the acquired skills and maintaining the new body weight, there are monthly 2-day return visits for the children. During the second day of the return visits the parents also take part in the treatment. Each return visit a different topic related to the treatment is being discussed with the children in sessions both with and without the parents attending. The topics discussed are: autonomy, the family, dealing with emotions, my body, self confidence and self image. The topics also help to look ahead and plan how to tackle possible problems in the future. During these return visits also problems encountered at home are discussed and used as educational examples.

Table 1 describes for both treatments in detail the number of appointments the children have with different professionals of the multidisciplinary team in different phases of the treatment. In addition to the contacts described in the table, two group coaches are always present during the day to provide ongoing learning and education. They can support the children in the learned behavior and observe the children and discuss possible difficulties. During the night there is always supervision of a night nurse. For more information on the different disciplines involved see Table 2.

**Table 1:** Details of treatment programs A and B in respect to visits to different health care professionals and treatment activities

	Treatment A				Treatment B			
**Disciplines/treatment parts**	**Phase 1 (weeks 1-6)**	**Phase 2 (weeks 7-18)**	**Phase 3 (weeks 19-26)**	Phase 4 (weeks 26-52)*	**Phase 1 (weeks 1-8)**	**Phase 2 (weeks 10, 12, 14, 16, 18), n = per visit**	**Phase 3 (weeks 20, 22, 24, 26), n = per visit**	**Phase 4 (weeks 26-52)**

**Exercise therapist**	4G	4G	3G	2G	3G	2G	2G	2G
**Cesar therapist**	1G	1G	1G		1G (from week 3 on)	-	Once this phase G	
**Dietician**	1I, 1G	1I, 1G	1I, 1G	Once this phase	Twice I in first 2 weeks	-	-	Once this phase
**Pediatrician**	Every other week 1I	Every other week 1I	Every other week 1I	-	Every other week 1I	-	Once this phase I	-
**Psychologist**	2I this phase	Once this phase I 1G education	1G education	-	2I this phase	Once (children and parents) this phase G	Once this phase I	-
**Nurse**	Twice this phase I	Twice this phase I	-	-	-	-	-	-
**Laboratory**	Once I this phase	-	Once I this phase	Once I this phase	Once I this phase	-	Once I this phase	Once I this phase
**Social worker (parents)**	1G, 2I this phase	1G	1G	1G	1G	1G	1G	1G
**Parents course (parents)**	Twice this phase G	3 times this phase G	Once this phase G	-	8 times this phase G	Twice this phase G	-	-
**TTV**	-	-	Once this phase I, max 1 week	-	-	-	-	-
**Education/group-activity children**	-	-	-	1G	2 to 4G	2 to 4G	2 to 4G	3G
**Education/group activity/training parents and children**	-	-	-	1G	1G	6 times this phase G	5 times this phase G	2G

G = group session

I = Individual session

**Table 2:** Explanation of different disciplines and treatment parts

Disciplines/treatment parts	Explanation of disciplines/treatment parts
**Parents course (parents)**	A course of several meetings for the parents in which different topics related to the treatment are being discussed under supervision of the social worker, such as making choices or handling temptations.

**TTV**	Temporarily therapy leave. A period with a maximum of 1 week in which the children of treatment A go home during the inpatient period to practice learned behavior in a normal setting during weekdays.

**Education/group-activity children**	Several topics related to the treatment are being discussed with the children under supervision of the group coaches such as motivation, bullying and self image Activities with the children are supervised by the group coaches. This can be something active like sport games or something to put an educational topic into practice.

**Education/group activity/training parents and children**	The educational sessions for both children and the parents together are always supervised by the group coaches and a social worker. Several topics related to the treatment are being discussed and occasionally the pediatrician or psychologist or dietician visits the session to discuss a certain topic. Activities with the children and their parents supervised by the group coaches and social worker. This can be something active like sport games or something to put an educational topic into practice.

### Measurements

Primary outcome measure is (SDS) BMI. Secondary outcomes are quality of life and cardiovascular risk factors like blood pressure, bodily circumferences, serum lipids, glucose levels and insulin levels. Eating behavior and physical activity are also assessed. The EuroQoL (EQ-5D) is used to measure quality of life and to calculate Quality Adjusted Life Years (QALYs) over the follow-up period of 24 months. Measurements will be done at four points in time: at baseline (start of treatment) and after 6, 12 and 24 months.

### Height, weight and circumferences

Height is recorded with a Holtain stadiometer fixed on the wall with an accuracy of 1 mm. The stadiometer is calibrated before every first measurement. Height is recorded three times of which the average is calculated. Weight is measured in light clothing without shoes and recorded with a calibrated SECA digital weight chair that has a limit of 230 kg and an accuracy of 0.005 kg.

Weight and height are used to calculate BMI (weight in kilograms divided by the square of height in meters). The degree of overweight is quantified using Cole's least mean square method, which normalizes the BMI's skewed distribution and expresses BMI as SDS-BMI [19]. These calculations are performed using (http://www.growthanalyser.org; version 3.5, program by "Stichting Kind en Groei", downloaded in July 2010). The data from the fourth Dutch growth study among children of 1997 are used as reference. The SDS-BMI indicates how many standard deviations a measurement is above or below the median of the distribution. Circumferences of the neck, waist, abdomen (WHO as well as maximum) and hip are measured with a tape measure. The participant has to stand up straight (in underwear) with the arms alongside the body and the feet in resting position. The circumferences are measured with an accuracy of 1 mm.

### Blood pressure

Blood pressure is measured with a digital blood pressure monitor (Heine, type Gamma E60). A cuff size with a width of two-third of the upper arm length is used that completely covers the arm circumference. For most participants a 17 cm cuff size (CAO2, arm circumference 33-41 cm) is used. If necessary, a 14 cm cuff size (CAO1, arm circumference 22 32 cm) is used. Blood pressure is measured in sitting position after sitting still for at least 5 minutes. Blood pressure is measured three times. For the analyses, the averages of the three systolic blood pressure and diastolic blood pressure readings are used.

### Bio-electrical impedance spectroscopy

Bio-electrical impedance spectroscopy (BIS) measurements are conducted with a Body Impedance Analyzer BIA 101/s (Akern-SRL Systems by EQUIP Medikey BV). Two current electrodes (tetra-polar electrodes (3 M red Dot AG/AgCl)) are placed at the dorsal surfaces of the hand and foot on the distal portion of the second metacarpal and metatarsal, respectively. Two detector electrodes are placed at the posterior wrist between the styloid processes of the radius and ulna and at the anterior ankle between the tibial and fibular malleoli. The resistance (Ohm) is used in the analysis to determine fat mass (FM) and fat free mass (FFM). The equation used for the children and adolescents in this study for percentage body fat (%BF) is the adjusted Kushner equation {Wt-[0.59(Ht2/R)+0.065(Wt)+0.04]/[0.754(Wt)]} x 100 [20]. This equation is adjusted and validated by Newton et al [21].

### Blood measurements

After an overnight fast, blood samples are obtained to measure lipid spectrum (cholesterol, HDL-cholesterol, LDL-cholesterol, triglycerides) liver function (y-GT, ALAT, ASAT) and C-reactive protein (CRP), haemoglobin, hematocrite, MCV, ferritin and HbA1C. In patients with a triglyceride level ≤ 4.5 mmol/l, LDL-cholesterol is calculated using the formula of Friedewald. Since this calculation is unreliable in patients with a triglyceride level > 4.5 mmol/l, LDL-cholesterol is measured directly in these patients using a Roche Cobas 6000 chemical analyzer. The method for this direct measurement is an enzymatic reaction with the transformation of LDL-cholesterol in a color product. High sensitive CRP measurement is performed turbidimetrically with a Roche Cobas 6000 chemical analyzer. To determine glucose tolerance and insulin resistance, the participants are given glucose, in a dose of 1.75 g per kilogram of body weight (up to a maximum of 75 g) orally. Blood samples are obtained at 0 and 120 minutes for the measurement of glucose and insulin levels. In accordance with the American Diabetes Association guidelines, impaired fasting glucose is defined as a fasting plasma glucose level between 5.6 - 6.9 mmol/l and impaired glucose tolerance as a 2-h postload glucose level in the oral glucose tolerance test (OGTT) between 7.8-11.0 mmol/l. Diabetes is defined as a fasting plasma glucose level of ≥ 7.0 mmol/l or a 2-h postload glucose level in the OGTT of ≥ 11.1 mmol/l [22].

### Quality of life

Quality of life is measured using the EQ-5D [23]. The EQ-5D questionnaire contains a descriptive system of health related quality of life consisting of five dimensions (mobility, self-care, usual activities, pain/discomfort, and anxiety/depression). For each dimension there are three levels of severity (no problems/some or moderate problems/extreme problems). A standard vertical 20 cm visual analogue scale is also included to measure an individual's direct valuation of their current health-related quality of life state. Utilities will be estimated using the Dutch tariff [24]. Quality Adjusted Life Years (QALYs) will be calculated by multiplying the utilities with the amount of time a participant spent in a particular health state. Transitions between health states will be linearly interpolated.

### Cost measures

Cost calculations will be based on actual resource use, using prospective data collection during 2 years (the 12 months intervention period and the 12 months follow-up) through cost diaries. The costs of the interventions (inpatient care and recurrent return visits of 2 days) will be estimated using a bottom-up approach. Other costs include direct healthcare costs (e.g. costs of visits to the general practitioner, internist, physiotherapist, inpatient period), direct non-healthcare costs (informal care provided by parents and legal guardians and travel costs), and indirect costs (costs related to loss of productivity of the parents and legal guardians). Cost diaries are formatted as calendars on which health care utilization can be registered. The calendars will be sent to the families every 3 months. After 3 months the calendars will be returned by the parents, using stamped envelopes, also if there has been no health care utilization during that period. In case a calendar is not returned, use of health care will be inventoried by telephone calls and/or e-mails.

If available, standard costs recommended by the Dutch Health Insurance Council will be used to value resource use [25]. Medication costs will be valued using prices of the Royal Dutch Society of Pharmacy [26].

### Sample size calculation

Based on a previous study comparing intensive inpatient treatment with an ambulatory treatment in the same treatment centre it is calculated that 40 participants in both intervention groups are sufficient to detect a 0.5 SDS-BMI difference between the two groups after one year (with α = 0.05 and power = 0.8). Based on earlier experience in the same setting and with similar participants it is feasible to recruit these numbers of participants.

### Statistical analyses

An intention-to-treat analysis will be performed. A per protocol analysis will also be performed after careful description of correlates of non-adherence and dropout.

To take into account the repeated measurements over time, we will use generalized estimating equations for panel data analysis, also known as cross-sectional time-series analysis, with the use of the Stata software XTGEE command; this will allow us to account for the non-independence of repeated measurements of the same bio-indicator in the same participant over time. Linear regression will be applied for continuous outcomes and logistic regression for dichotomous outcomes. We will use age, sex, time point, and intervention group as explanatory variables in our models.

An economic evaluation from a societal perspective will be conducted alongside the randomized controlled trial. Multiple imputation will be used to impute missing cost and effect data. Five imputed data sets will be created, each of which will be analyzed separately. The results of these five analyses will be pooled using Rubin's rules [27].

Costs generally have a highly skewed distribution. Therefore, bootstrapping with 5000 replications will be used to estimate "approximate bootstrap confidence" (ABC) intervals around cost differences [28,29]. Incremental cost-effectiveness and cost-utility ratios will be calculated by dividing the difference in total costs between the groups by the difference in SDS-BMI and QALYs, respectively. Non-parametric bootstrapping will also be used to estimate the uncertainty surrounding the incremental cost-effectiveness and cost-utility ratios (5000 replications). The bootstrapped cost-effect pairs will be plotted on a cost-effectiveness plane (CE plane) [30] and used to estimate cost-effectiveness acceptability curves (CEA curves). CEA curves show the probability that the intervention is cost-effective in comparison with usual care for a range of ceiling ratios. The ceiling ratio is defined as the amount of money society is willing to pay to gain one unit of effect [31].

## Discussion

This paper presents the design of a randomized controlled trial comparing the cost effectiveness of two intensive one-year inpatient treatments to each other and to usual care for severely obese children and adolescents. The study will not only provide insight in the effects of 1 year treatment, but also the maintenance one year after the end of the treatment. Studies regarding the effectiveness and cost-effectiveness of treatment programs (with or without an inpatient period) for obese children and adolescents are relatively rare, especially for the severely obese [13]. Kelly and Kirschenbaum reviewed published studies on inpatient treatment for childhood and adolescent obesity [12]. Their review showed that programs containing an inpatient period had better weight loss and subsequent weight maintenance compared to the outpatient treatments. The rates of attrition were also lower in the inpatient treatment groups. Our study can add important knowledge on the usefulness of inpatient treatment in severely obese children and adolescents.

An important strength of this study is the randomized controlled trial design. Another strength is that both young children (8-13 years of age) and adolescents (13-18 years of age) will be included in this study, since obesity is an important health problem in these age groups. This study provides information on the effectiveness and feasibility of the intervention in both age groups that differ in environmental and individual factors associated with obesity, such as the role of the parents and peers. Also participants of different ethnic groups and from rural and urban areas from different parts in the Netherlands will participate in the study. This will contribute to the generalizability of the outcomes to the general population of severely obese children and adolescents in the Netherlands. Moreover this study will provide information on the feasibility to implement the program on a larger scale and perhaps also in other settings such as regular hospitals.

A challenge of the study will be keeping the attrition rate to a minimum. The study population is expected to have a relatively low socioeconomic status (SES) and to present relatively many psychological problems and socially complicated family structures. This can interfere with adherence to the treatment program. The psychological characteristics of the patients and their parents will be described in a separate study. Another difficulty will be the recruitment of participants, because of the extended inpatient periods. Especially in the group of children aged 8-13, we expect problems with recruitment because an inpatient period of 2 or 6 months imposes a very heavy burden on families and children. Also, because recruitment is nationwide, part of the parents will have to travel quite a lot, since their active participation and frequent presence during treatment is required. This can lead to high work absenteeism for the parents or loss of vacation days which will also be measured.

For the calculation of %BF the adjusted equation by Kushner et al is used [20], since the original equation by Kushner et al is developed to calculate total body water and is therefore not applicable. We have chosen for this equation after careful consideration as there are more suitable equations to calculate %BF specifically developed for obese children and adolescents such as the equations of Lazzer et al [32] and Schaeffer et al[33]. However we are limited in our choice because only resistance (R) is recorded. Cost calculations will be based on actual resource use; therefore prospective data collection through cost diaries during 2 years is used. However, the risk of this type of data collection is drop out; the longer the participant are followed, the higher the chance of loss to follow up. Also, bias can be introduced with this type of data collection, since it is based on self reporting. People can forget to note absenteeism or visits to health care professionals, even with the use of the cost diaries. By prospectively collecting data we hope to get valid estimates of health care utilization.

The major cost savings as a result of treatment are expected to come about much later in the lives of these children, the effects on productivity, absenteeism, disease incidence and use of medical care potentially may have an effect after decades. To be able to predict such cost savings, this short term cost-effectiveness analysis is too limited because this study is based on actual resource use, using the actual costs of the interventions, direct healthcare costs, direct non-healthcare costs and indirect costs for the period of 2 years. In the long term the cost savings will be much higher than in this short term of 2 years, therefore these interventions are investments in the long term. To make models to predict these long term cost savings is risky, because the long term effects of such interventions are not known.

Despite the challenges mentioned above, the results of the study will offer valuable information to health care professionals as well as policy makers regarding treatment of severely obese children and adolescents.

## Competing interests

The authors declare that they have no competing interests.

## Authors' contributions

All authors contributed to the design of the study. OB, SM and JH were responsible for acquisition of data. SM prepared the initial draft of the manuscript. All authors contributed to the writing of the manuscript and approved the final version of the manuscript.

## Pre-publication history

The pre-publication history for this paper can be accessed here:

http://www.biomedcentral.com/1471-2458/11/518/prepub
